# DNA methylome variation in a perinatal nurse-visitation program that reduces child maltreatment: a 27-year follow-up

**DOI:** 10.1038/s41398-017-0063-9

**Published:** 2018-01-10

**Authors:** Kieran J. O’Donnell, Li Chen, Julia L. MacIsaac, Lisa M. McEwen, Thao Nguyen, Katherine Beckmann, Yuecai Zhu, Lawrence Ming Chen, Jeanne Brooks-Gunn, David Goldman, Elena L. Grigorenko, James F. Leckman, Josie Diorio, Neerja Karnani, David L. Olds, Joanna D. Holbrook, Michael S. Kobor, Michael J. Meaney

**Affiliations:** 1 The Ludmer Centre for Neuroinformatics and Mental Health, Douglas Hospital Research Centre and Sackler Program for Epigenetics and Psychobiology, McGill University, Montreal, QC H4H1R3 Canada; 2 Canadian Institute For Advanced Research, Child and Brain Development Program, Toronto, M5G 1Z8 Canada; 3 Singapore Institute for Clinical Sciences (SICS), Agency for Science Technology and Research (A*STAR), Singapore, 119077 Singapore; 4 Centre for Molecular Medicine and Therapeutics, Child and Family Research Institute, and Department of Medical Genetics, University of British Columbia, Vancouver, BC V5Z 4H4 Canada; 5 National Center for Children and Families, Teachers College, Columbia University, New York, NY 10027 USA; 6 College of Physicians & Surgeons, Columbia University, New York, NY 10032 USA; 7 Laboratory of Neurogenetics, National Institute on Alcohol Abuse and Alcoholism (NIAAA), National Institutes of Health (NIH), Rockville, MD 20852 USA; 8 Department of Psychology, University of Houston, Houston, TX 77204 USA; 9 Departments of Molecular and Human Genetics and Pediatrics, Baylor School of Medicine, Houston, TX 77030 USA; 10 Department of Psychology, St. Petersburg State University, St. Petersburg, 199034 Russia; 11 The Child Study Center and the Departments of Psychiatry, Pediatrics, and Psychology, Yale University, New Haven, CT 06519 USA; 12 Department of Pediatrics, School of Medicine, University of Colorado, Aurora, CO 80045 USA

## Abstract

This study reveals the influence of child maltreatment on DNA methylation across the genome and provides the first evidence that a psychosocial intervention program, the Nurse Family Partnership (NFP), which targets mothers at risk for abusive parenting, associates with variation in the DNA methylome in adult offspring. The 188 participants were born to women randomly assigned to control (*n* = 99) or nurse-visited intervention groups (*n* = 89) and provided blood samples and a diagnostic interview at age 27 years. Interindividual variation in the blood DNA methylome was described using principal components (PC) scores derived from principal component analysis and showed that the NFP program (PC10: *p* = 0.029) and a history of abuse/neglect (PC1: *p* = 0.029, PC2: *p* = 0.009) significantly associated with DNA methylome variation at 27 years of age independent of gender, ancestry, cellular heterogeneity, and a polygenic risk index for major psychiatric disorders. The magnitude of the association between child maltreatment and DNA methylation was reduced when accounting for lifestyle factors, including smoking. These findings reflect the sustained impact of both childhood adversity as well as intervention programs that target such adversity on the epigenome but highlight the need for prospective longitudinal studies of DNA methylome variation in the context of early intervention programs.

## Introduction

Childhood adversity increases the risk for most forms of non-communicable chronic diseases^1^. Childhood adversity also associates with chemically stable, epigenetic modifications, such as DNA methylation^2–4^, which may then maintain the environmental effect on genomic function and health^3^. Genome-wide DNA methylation (the DNA methylome) is dynamic across development^5,6^ and altered by early social experience, as shown in peripheral cells as well as in postmortem brain samples^7,8^. These findings suggest the DNA methylome may help identify those affected by early life adversity and facilitate precision medicine initiatives in psychiatry.

One study to date has provided some evidence that a psychosocial intervention may moderate the effects of parental depression on features of the DNA methylome such as the epigenetic age^9,10^. However, data from programs of early intervention, with establishing clinical efficacy, are lacking. Such evidence is required to evaluate the clinical utility of the DNA methylome for personalized approaches in the fields of perinatal psychiatry and child/adolescent mental health.

This report describes the results of a unique 27-year longitudinal study examining the influence of child abuse/neglect (CAN) and maternal participation in the Nurse Family Partnership (NFP) program, a psychosocial intervention program that specifically targets maternal health and mother–child interactions^11^, on genome-wide DNA methylation. The NFP reduces CAN and improves neurodevelopmental outcomes^12,13^. We hypothesized that variation in genome-wide DNA methylation would associate with both CAN and the NFP intervention.

## Methods

### Participants

We used the original NFP trial sample (*N* = 400) conducted with a primarily Caucasian sample (89%), which has been described in detail previously^12,13^. Primiparous women were recruited with one or more of the following risk factors: young age (<19 years), unmarried, or low socioeconomic status (SES). Women were randomly assigned to control or nurse-visited groups. Treatment groups received nurse visits during pregnancy or to age 2 years postpartum. Nurses promoted maternal health behaviors, parental care, and personal development. All nurse-visited women were combined to form a single intervention group given comparable program effects on adolescent antisocial behavior through age 15 years^13^. Approval for this study was obtained from the Institutional Review Board (University of Colorado) protocol #04-0002.

Offspring (mean age 27.4, SD = 0.7 years) of women from the control (*n* = 99) and intervention (*n* = 89) groups provided blood samples and completed the computerized diagnostic interview schedule^14^ to assess (1) major depression, (2) generalized anxiety, (3) PTSD, (4) antisocial personality/conduct disorder and (5) substance abuse. We created a summary variable reflecting the total number of psychiatric diagnoses. Participants' history of CAN from birth to age 15 years was detailed using substantiated reports from Child Protective Services records and converted to a binary variable (0 = no indication of CAN, 1 = substantiated case of CAN).

### Genome-wide DNA methylation analyses

Biosamples from the adult offspring of mothers enrolled in the NFP and DNA methylation was described using a genome-wide platform^15^. First, genomic (gDNA) was extracted from blood^16^, bisulfite converted using the EZ-DNA Methylation Kit (Zymo Research), and hybridized on the Illumina Infinium HumanMethylation450K BeadChip (450K array) according to the manufacturers' protocol. The experiment was run in three batches with samples randomized across chip and technical replicates (*n* = 19) included for quality control of chip and batch effects. We identified a chip and batch effect, which was corrected using ComBat^17^. One sample failed quality control, e.g., low call rate^18^ (*n* = 1) and was removed, resulting in a final pool of 187 (control *n* = 98, nurse-visited *n* = 89) samples. The 450K array provides quantitative data on DNA methylation at >480,000 CpGs across the genome. We focused our analysis on autosomal CpGs showing interindividual variation in DNA methylation (range of ≥10%), thus denoted as variably methylated CpGs (vCpGs: *n* = 178,964). vCpGs were subjected to principal component analysis (PCA) within Array Studio (OmicSoft). PCA is a data reduction strategy that describes the variability in a dataset using discrete, independent components^19^. Principal component 1 (PC1) is the linear function that describes the greatest amount of variability in the data, followed by PC2, etc.

### Transcription factor interaction and enrichment analyses

We used MetaCore+MetaDrug v6.23.67496 (GeneGo) and Enrichr^20^ for gene ontology and transcription factor interaction analysis. P-values for transcription factor interaction analyses were determined using hypergeometric intersection (Metacore) or a modified Fisher’s exact test (Enrichr).

### PCA enrichment analyses

We extracted PC loadings for each vCpG and each of the top 10 PC scores. We identified vCpGs (1) most strongly associated with a given PC score (defined as loading score within top 10%) and (2) nominally associated (*p* < 0.05) with CAN-associated vCpGs or NFP treatment group. We performed Enrichr analyses on the resultant vCpGs associated with a PC score and a predictor of interest (e.g., CAN or treatment group).

### Epigenetic age acceleration

We estimated epigenetic age on the basis of DNA methylation of 353 ‘clock’ CpGs as previously described^10^. Epigenetic age acceleration, reflecting a discrepancy between chronological age and epigenetic age estimates, was given by the residual from a linear model that regresses epigenetic age on chronological age.

### Population structure and polygenic risk index for psychiatric disorders

Genotype can influence variation in DNA methylation^21^. We tested whether group differences across the DNA methylome were confounded by population structure or genetic liability for psychiatric illness. First, we described allele frequencies at 248,648 autosomal single-nucleotide polymorphisms (SNPs) using a genome-wide platform (PsychChip, Illumina) according to the manufacturers' guidelines. We removed SNPs with a low call rate (<95%) and minor allele frequency (<5%) and used PCA to describe population structure within the NFP cohort^22^. Two PCs best described the population structure within this cohort (see Supplemental Figure S1) and were included in all subsequent analyses.

We performed imputation using the Sanger Imputation Service^23^ resulting in 24,412,593 SNPs with an info score >0.80 and posterior genotype probabilities >0.90. Next, we generated a polygenic risk score (PRS) for each participant within our dataset using a custom script implemented in Python and Apache Spark (Chen et al., under review: see https://github.com/MeaneyLab/PRSoS) informed by findings from the large independent dataset from the Psychiatric Genomics Consortium (PGC) encompassing five major psychiatric disorders^24,25^. We selected SNPs that associated with a range of mental disorders in the PGC ‘discovery dataset’ at a variety of *p*-value thresholds: *p* < 0.5–0.001 and generated corresponding PRS as previously described^25^.

### Data analysis

All multivariate models of PC scores included gender, age, number of psychiatric diagnoses, maternal education (as an index of early life socioeconomic status), and estimates of the proportions of major cell types found in whole blood derived from methylation data using a well-established reference-based algorithm^26^. We also excluded all non-specific and SNP-disrupted probes^27^ in our regression models to identify vCpGs associated with a history of CAN. We used a two-sample one-tailed Kolmogorov–Smirnov goodness of fit hypothesis test (pkstest) to examine *p*-value distributions, as test of the null (unskewed/flat) distribution. A distribution with pkstest <0.001 was considered a significantly skewed distribution.

## Results

There were no significant group differences in maternal characteristics at baseline, participant gender, or self-reported ethnicity between those included in our analysis at age 27 years and the remainder of the Elmira NFP cohort (Table 1). Participants born to women from the NFP control groups and those with a history of child maltreatment were overrepresented in our biosample cohort. There were no group differences in the prevalence of child maltreatment within this subsample (*p* = 0.460). Those providing biosamples experienced more mental health problems than the remainder of the Elmira cohort at age 27 years (*p* = 0.005). There was no difference in the total number of diagnoses at age 27 years between the nurse-visited group and controls (*p* = 0.401) in the biosample cohort. Likewise, there was no difference in psychiatric diagnoses between those reporting a history of maltreatment and the remainder of the group (*p* = 0.457).

**Table 1:** Participant and maternal characteristics of 27-year follow-up of Elmira Nurse Family Partnership cohort

Elmira NFP 27-year follow-up		
	450K Cohort, *N* = 187	Remainder of the NFP, *N* = 213
Participant characteristics		
Gender (% of female)	52.9%	47.9%
Treatment group (% of nurse-visited)	47.6%*	59.6%
Ethnicity (% Caucasian)	84.5%^#^	75%
% missing	na	30.3%
Child abuse/neglect (%)	27.3%*	15.5%
% missing	6%	33%
No. of psychiatric diagnoses (SD)	1.07 (1.19)**	0.71 (0.88)
% missing	0.5%	42%
Maternal characteristics		
Ethnicity (% Caucasian)	91.4%^#^	85.9%
Age in years (SD)	19.2 (3.0)	19.6 (3.3)
Education (SD)	11.2 (1.4)	11.2 (1.7)
Hollingshead score (SD)	54.0 (15.6)	55.2 (20.9)
Marital status (% unmarried)	62.6%	60.6%

Mean and standard deviation (SD) or percentages are provided and group differences tested using Student’s *t*-tests or chi-squared tests where appropriate. ^#^ *p* < 0.10, *p < 0.05, **p < 0.01, na = not applicable

### NFP treatment group, CAN associate with DNA methylome variation

**Fig. 1:**
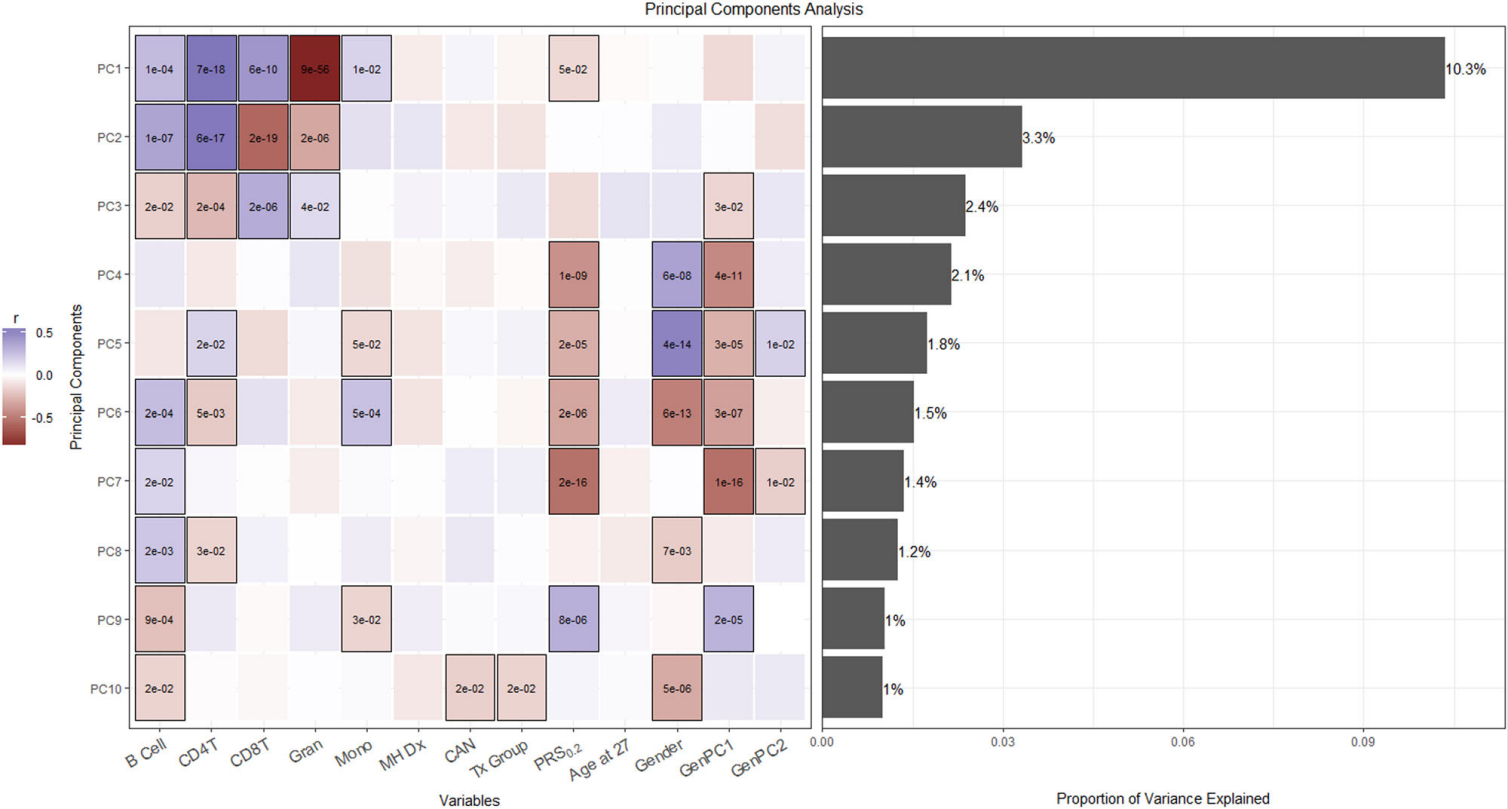
Bivariate association between predictors of interest and DNA methylome variation Heatmap describes the unadjusted bivariate association (Pearson’s *r*) between predictors of interest and the first 10 principal component (PC) scores from a principal component analysis (PCA) of variably methylation CpGs. Bars (right panel) represent the percentage variance accounted for by each component. p-Values are provided for significant (*p* < 0.05) associations. *CAN:* child abuse and neglect, *Tx Group:* Nurse Family Partnership group (control vs. nurse-visited), *GenPC1 and GenPC2:* PC scores derived from PCA of genetic variation, *MH Dx:* psychiatric diagnoses

We used PCA of vCpGs to generate PC scores for the first 10 PCs for each participant. The first 10 PCs accounted for 26% of the variance in DNA methylation. Figure 1 shows the unadjusted bivariate associations between the first 10 PC scores and the variables considered in our analyses. As expected, cell count estimates were significantly associated with PC1–3, which explain the largest proportion of the variance in DNA methylation.

**Fig. 2:**
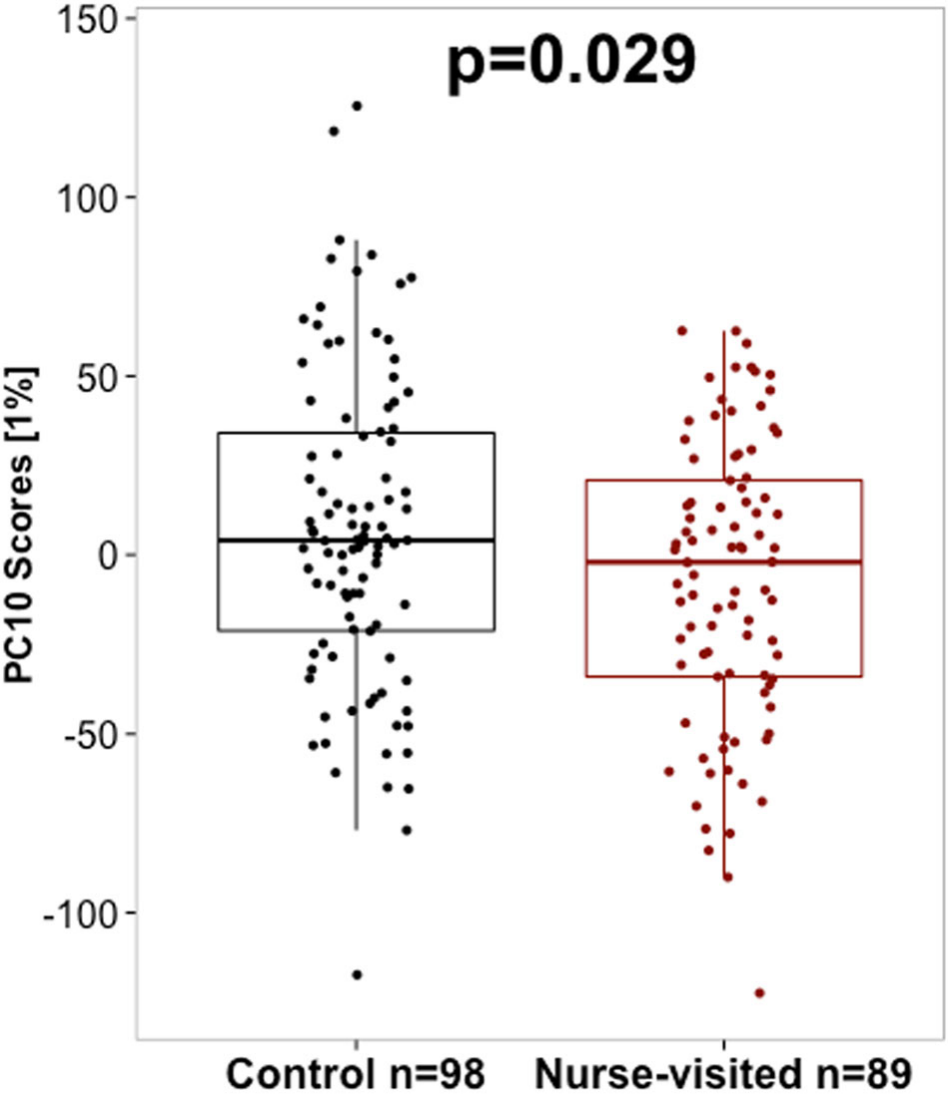
The Nurse Family Partnership (NFP) intervention associates with DNA methylome variation Principal component 10 (PC10) scores (*y* axis) were significantly higher in the control (black) vs. nurse-visited (red) group. p-Values from ANOVA models controlling for cell type, age at time of biosampling, population stratification index by two principal component scores, gender, maternal education, psychiatric diagnoses recorded at age 27 years, a cross-disorder polygenic risk score, and maltreatment history

Next we used analysis of variance (ANOVA) to test the association between the maternal NFP treatment group and DNA methylome variation. Maternal treatment group (control vs. nurse-visited) showed a significant association with PC10, which accounted for 1.0% of the variance in DNA methylation. The association between PC10 and NFP treatment group survived adjustment for early life SES, gender, population stratification, age at time of biosampling, cell type, psychiatric diagnoses, and maltreatment history (*p* = 0.029; Fig. 2). The association between maternal NFP participation and PC10 was not moderated by maltreatment in the offspring (*p* for interaction = 0.174). Enrichr analysis of CpGs that associated with PC10 and maternal participation in the NFP revealed enrichment for biological processes related to synaptic function, neurogenesis, and peripheral nervous system development (see Supplemental Table S1).

Since the NFP treatment specifically targets parental care and thus diminishes the rate of childhood adversity, we predicted an association between CAN and DNA methylation. Indeed, our ANOVA models revealed multiple significant associations between CAN and variation in the DNA methylome. CAN was significantly associated with both PC1 (*p* = 0.029) and PC2 (*p* = 0.009), which accounted for 10.3 and 3.3% of the variance in DNA methylation, respectively. Enrichr analysis of vCpGs associated with PC1 and maltreatment history revealed a trend-level enrichment for biological processes related to protein phosphorylation and immune cell activation/differentiation. CpGs that associated with PC2 and maltreatment history revealed significant enrichment for processes related to transcription factor regulation and transcription (see Supplemental Table S2).

**Fig. 3:**
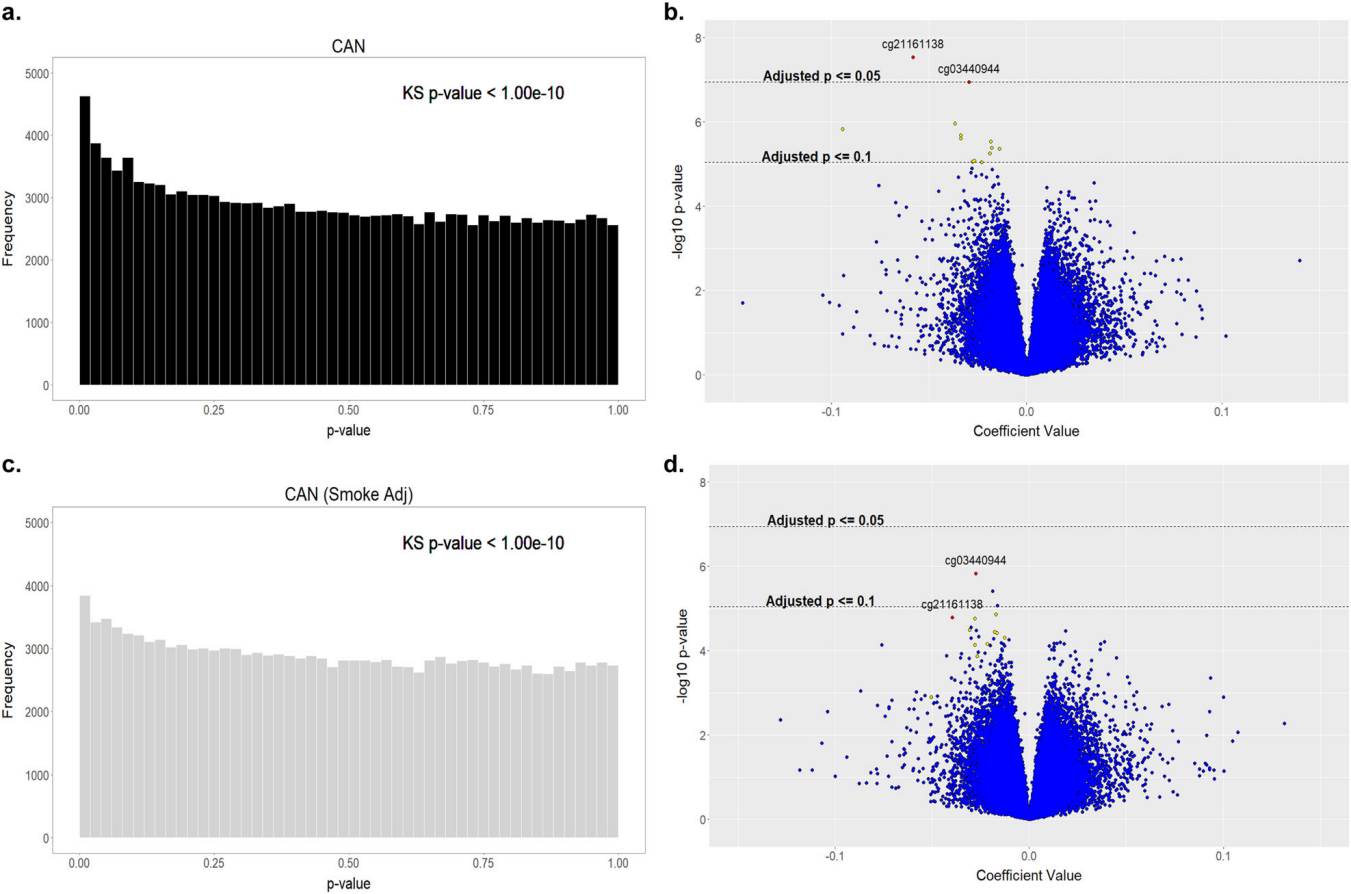
Child abuse/neglect (CAN) associates with DNA methylome variation at 27 years of age The p-value distribution **a** and volcano plot **b** of variably methylated CpGs (vCpGs) associated with CAN highlight vCpGs associated with CAN at Benjamini–Hochberg False Discovery Rate (BH-FDR) corrected *p* < 0.05 (red points) and *p* < 0.10 (yellow points) before but not after adjustment for smoking **c**, **d**. See Supplementary Table S3 for rank order of vCpGs across both models

Linear regression analysis of CAN and vCpGs revealed a highly right-skewed *p*-value distribution (Fig. 3a) that significantly deviated from that expected under the null hypothesis (pkstest < 1.00E-10). There were 10,481 vCpGs nominally (unadjusted *p* < 0.05) associated with CAN, with two vCpGs passing Benjamini–Hochberg False Discovery Rate correction (BH-FDR-adjusted *p* < 0.05; Fig. 3b). One of these CAN-associated vCpGs (cg21161138) resided in *AHRR*, which encodes the Aryl-hydrocarbon Receptor Repressor (AHRR). *AHRR* DNA hypomethylation (including cg21161138) consistently associates with smoking^28,29^. Likewise DNA methylation at cg03440944, located in *C7orf40*, associates with smoking^30,31^. We tested whether participants with a history of maltreatment reported greater smoking at time of biosample collection. Indeed, we found a significant association between CAN and current smoking that is consistent with previous reports linking child maltreatment to increased health-risk behaviors^32^. Thus 75% of participants with a history of CAN were current smokers compared with 41% of pariticipants from the non-abused group (*χ* ^2^ = 16.12, *p* = 5.8E-05). The association between smoking and CAN with *AHRR* DNA methylation was confirmed using an independent analysis of DNA methylation by pyrosequencing (Supplemental Figures S2&S3). These analyses not only verified the DNA methylation data from the 450K array but also revealed a similar pattern of association between smoking/CAN with two additional CpGs located within *AHRR*. Current smoking also predicted an increased abundance of CD8-positive T cells in blood samples at age 27 years (*B* = 0.006, *β* = 0.093, *p* = 0.001), providing an additional source of biological variance in DNA methylation. Nevertheless, controlling for smoking in our regression model marginally reduced the number of CAN-associated vCpGs (10,344–9120) but significantly reduced the effect size (Cohen’s *d* = 0.82–1.00 to *d* = 0.58–0.70) of CAN on DNA methylation of vCpGs within *AHRR C7orf40* (all BH-FDR-adjusted *p* > 0.05; Fig. 3c). See supplemental Table S3 for top ranked CAN-associated vCpGs.

Next we tested whether current smoking confounded our ANOVA findings. The association between CAN and PC1 was reduced to trend level (*p* = 0.082) but controlling for smoking did not substantively change the association between CAN and PC2 (*p* = 0.011). Likewise, the association between maternal participation in the NFP and PC10 was relatively unchanged (*p* = 0.038) following adjustment for smoking.

### CAN vCpGs associate with steroid receptor hormones

Next we carried out an Enrichr analysis of vCpGs associated with child maltreatment after adjustment for confounders including smoking. This analysis uses chromtin immunoprecipitation–sequencing across multiple human cell lines to identify genes regulated by specific transcription factor. Child maltreatment-associated vCpGs were enriched in genes regulated by a number of transcription factors, including multiple steroid hormone receptors, notably the androgen receptor, the glucocorticoid receptor (*NR3C1*), estrogen receptor alpha (*ESR1*), and estrogen receptor beta (*ESR2*; See Supplemental Table S4). The enrichment of maltreatment-associated vCpGs within genes regulated steroid hormone receptors was further supported by analyses in Metacore (see Table S5).

### Polygenic risk for common mental disorders associates with current mental health

We identified a cross-disorder PRS at a *p*-value threshold *p* ≤ 0.20 (Cross-PRS_0.2_) that associated with the number of current psychiatric diagnoses in the NFP biosample cohort (*r* = 0.144, *p* = 0.049). There was no significant between-group differences in Cross-PRS_0.2_ as a function of child maltreatment (*p* = 0.605) or maternal participation in the NFP program (*p* = 0.716).

### A PRS for psychiatric disorders associates with DNA methylation

Unadjusted bivariate analyses showed that Cross-PRS_0.2_ was significantly associated with multiple PC scores (see Fig. 1). Linear regression analysis revealed a significantly skewed *p*-value distribution (*p* < 1.0E-10; see Supplemental Figure S4). Taken together, these findings suggest that the Cross-PRS_0.2_ associates with a significant proportion of variance in the DNA methylation. Next, we tested whether the vCpGs nominally associated with child maltreatment overlapped with those associated with the Cross-PRS_0.2_. They were not (*p* = 0.117; see Supplemental Figure S5). Likewise, controlling for the Cross-PRS_0.2_ did not substantively change the findings from our ANOVA models. Maternal participation in the NFP treatment remained significantly associated with PC10 scores (*p* = 0.049), while CAN was associated with PC1 (*p* = 0.079) and PC2 (*p* = 0.009) after adjusting for the Cross-PRS_0.2_.

### Supplementary analysis

#### CAN does not associate with epigenetic age acceleration

Chronological age and estimated epigenetic age were moderately correlated in this cohort (*r* = 0.37, *p* = 2.80E-07). We found no significant association between measures of epigenetic age acceleration and history of child maltreatment (*p* = 0.530), maternal participation in the NFP program (*p* = 0.491), or the number of mental disorders recorded at age 27 years (*p* = 0.152).

## Discussion

In the context of a 27-year longitudinal study, we have described the persisting influence of the early social environment on variation in DNA methylation across the genome. CAN was significantly associated with PC1 and PC2, which together accounted for ~14% of the interindividual variation in DNA methylation across the human genome in adulthood. These findings underscore the profound impact of childhood adversity on the human epigenome and are reminiscent of studies describing the impact of child abuse on DNA methylation of *NR3C1* ^8,33^, which codes for the glucocorticoid receptor, and *FKBP5*, which codes for a glucocorticoid receptor chaperone^7^. The association between childhood adversity and the methylation status of these genes is apparent in peripheral cells^33^ and associated with concurrent psychopathology^34^. This effect of childhood adversity is also consistent with our finding of a detectable influence of the NFP psychosocial intervention, which specifically targets CAN, apparent in a genome-wide methylation analysis performed 25 years following the termination of the intervention.

CAN-associated vCpGs showed significant enrichment within genes that interact with a number of transcription factors, including multiple steroid hormone receptors. This finding suggests environmentally regulated transcription factor signaling may activate the remodeling of DNA methylation. This idea is consistent with previous work that associates CAN with altered methylation of CpGs within or proximal to glucocorticoid response elements^7^. Transcription factor-initiated remodeling of the DNA methylome has emerged as one of the mechanisms in support of the environmental epigenetic hypothesis linking variation in the early social environment to sustained changes in DNA methylation^7,35^ and increase risk of adverse health outcomes^36^. This mechanism is conserved across a number of transcription factors, including *NR3C1* ^7^ and *Esr1* ^37^. The environmental regulation of factors, such as steroid hormones, that act on common receptor systems in a wide range of cell types could explain potential cross-tissue concordance for the effects of CAN on variation in DNA methylation.

The association between CAN and the DNA methylation of *AHRR* suggests that childhood adversity might have direct as well as indirect effects on the epigenome by influencing health-risk behaviors, which in turn may influence biological factors, e.g., cell-type proportions. While this pathway must be directly confirmed, the findings reflect the potential complexity of associations between early childhood environments and adult phenotypes. Our findings do suggest that the cumulative direct and indirect effects of early intervention should be considered when evaluating treatment effects on variation in DNA methylation, a point that is especially important for epigenetic studies of developmental history and adult health outcomes. Likewise, our finding that current smokings predicts the proprotion of CD8-positive T-cells in whole blood further emphasises the need to consider cell type in studies of early adversity^38^.

Maternal NFP participation or history of CAN did not predict epigenetic age acceleration in this cohort. This is in line with a recent study which found that cumulative lifetime stress, rather than childhood trauma alone, predicted epigenetic age acceleration^39^. However, it should be noted that the age range in our cohort (3.9 years) is somewhat comparable to the error within the epigenetic clock predictor (3.6 years)^10^. In line with our data, Brody et al. report no direct association between a program designed to reduce harsh parenting and epigenetic age acceleration in a large sample of young adults^9^. However, the authors do note a moderating role of the intervention on parental depression-associated epigenetic age acceleration, with significantly greater age acceleration in the control group. Taken together with the results of our PCA, such findings suggest that intervention programs may influence features of the DNA methylome and could be of use for precision medicine efforts in child/adolescent health but further work is needed.

We do note that the association between maternal NFP program participation and offspring DNA methylation was small, accounting for <1% of the variance in the DNA methylome. However, the 450K array describes <3% of the DNA methylome and provides limited coverage of enhancer regions, which play an important role in transcriptional regulation and mental health^40^. Likewise, our analyses are based on those participants who provided biosamples at age 27 years, approximately half of the original Elmira NFP cohort, who were more likely to have experienced abuse and psychiatric disorder. It is possible that our findings were affected by the selective attrition evident at the 27-year follow-up (Olds et al., under review) and thus may underestimate NFP program effects on DNA methylome variation.

Our study benefits from the randomized controlled trial and longitudinal design of the NFP study and an objective measure of CAN (substantiated cases from Child Protective Services records). Indeed our findings may also underestimate the true effect of CAN on methylome variation due to unreported instances of maltreatment and the likelihood of at least some CAN contamination within our control group^41^. Likewise, our study benefits from the inclusion of paired genetic data as an important determinant of variation in DNA methylation. We used a broad index of genetic risk for multiple psychiatric disorders and report not a significant association with both the number of current psychiatric diagnoses and variation in DNA methylation. However, our PRS analyses provided little evidence of overlap between PRS- and child maltreatment-associated vCpGs. These findings are somewhat in line with those of Hannon et al., who report little overlap between CpGs associated with the presence of a diagnosis of schizophrenia and a PRS for the disorder^42^.

Limitations of our study include the lack of a complete blood count at the time of sample collection, which we addressed using a well-established computational method to ascertain cell type based on DNA methylation profiles^26^ and considered cell type in all of our analyses. However, we cannot rule out residual confounding by cell types not estimated by this method. Likewise, due to the unique nature of this 27-year study our sample size was relatively small and a comparable replication cohort was not available. The primary objective of this study was to examine how the early social environment shapes variation in the DNA methylome. We did not set out to perform an epigenome-wide association study of NFP program effects on DNA methylation and were not powered to do so (see Supplemental Table S6 and Table S7).

Another limitation is the cross-sectional nature of biological sample collection and the time from the termination of the NFP intervention at age 2 years to the sampling at 27 years. Our biosampling strategy also precluded an analysis of gene expression or other features of the epigenome, which limits our understanding of the functional consequence of variation in DNA methylation. As such, a causal analysis of the effects of the NFP or CAN on DNA methylation is not possible and these findings require replication, especially in light of our finding that child maltreatment associates with health-risk behaviors, which in turn predict variation in DNA methylation. A longitudinal, prospective study of the epigenome is needed to directly address these issues and is justified by our findings. These considerations notwithstanding, our analyses reveal a significant association between the maternal-focused NFP intervention and variation in genome-wide DNA methylation in the adult offspring. We initially hypothesized such a relationship based on the strong effects of the NFP intervention on the health and behavior of the offspring^12,13,43^.

The results reported here further document the impact of the social environment on the epigenetic signals that regulate genomic function and provide preliminary evidence for the impact of psychosocial early interventions on the epigenome. Prospectively designed cohorts incorporating longitudinal biosampling will clarify the relationship between early intervention and the DNA methylome and its predictive value for mental health outcomes.

## Electronic supplementary material

Supplementary Tables and Figures
