# Supplementary material for: DNA methylome variation in a perinatal nurse-visitation program that reduces child maltreatment: a 27-year follow-up
Source: Transl Psychiatry. 2018 Jan 10;8:15. doi: 10.1038/s41398-017-0063-9 (PMC5802588; doi:10.1038/s41398-017-0063-9)
Supplement: Supplementary file 1 — Supplementary Tables and Figures [file 41398_2017_63_MOESM1_ESM.docx]

**DNA methylome variation in a perinatal nurse-visitation program that reduces child maltreatment: a 27-year follow-up.**

**Supplementary Material**

**Table S1**: Enrichr analysis (biological processes) of vCpGs associated with PC10 and maternal participation in the nurse-family partnership (NFP).

**Table S2**: Enrichr analysis (biological processes) of vCpGs associated with PC1 or PC2 and history of child abuse/neglect (CAN).

**Table S3:** Child abuse/Neglect Associated vCpGs. The top 100 vCpGs from a linear regression analysis are provided. The model adjusted for cell-counts, gender, age at time of biosampling, population stratification, maternal education, psychiatric disorders and a polygenic risk score for psychiatric disorders. All p-values are unadjusted.

**Table S4:** Enrichr transcription factor enrichment analysis. Chromatin Immunoprecipitation sequencing datasets were accessed through Enrichr and enrichment for transcription factor binding examined within genes containing child abuse and neglect-associated vCpGs. Steroid-associated nuclear receptors are highlighted in bold.

**Table S5:** Metacore transcription factor enrichment analysis of variably methylated CpGs associated with child abuse/neglect. Bold text denotes steroid hormone receptors.

**Table S6:** Treatment group-associated vCpGs. The top 100 vCpGs from a linear regression analysis of maternal nurse family partnership treatment group and variably methylated CpGs are provided. The model adjusted for cell-counts, gender, age at time of biosampling, population stratification, maternal education, psychiatric disorders and a polygenic risk score for psychiatric disorders. All p-values are unadjusted.

**Table S7:** Effects size estimate and power calculation for detecting genome-wide significant associations between nurse family partnership participation and variation in DNA methylation.

**Figure S1:**  Principal component analysis of genetic data. Scree plot (left) shows the proportion of variance accounted for by each component. Principal component one (PC1) and PC2 best described the population structure in this cohort (markers are colour coded by self reported ethnicity).

**Figure S2:** Technical validation of *AHRR* DNA methylation. DNA methylation of *AHRR* as determined by the 450K array and pyrosequencing was highly correlated (left panel) and reproducible (right panel) across methods. Blue line (left panel) represents regression line, purple lines (right panel) represent the mean difference (solid line) and limits of agreement (dashed lines).

**Figure S3:** *AHRR* DNA methylation associates with child abuse/neglect (CAN) and smoking. The 204bp amplicon interrogated by pyrosequencing contained two additional CpGs 25bp and 23bp upstream of cg05575921 one of the top ranked variably methylated CpGs associated with CAN, respectively. All three CpGs showed a similar pattern of association with a history of CAN (a-c) and smoking (d-f). All p≤2.00E-06.

**Figure S4:** A polygenic risk score (PRS) for major psychiatric disorders associates with DNA methylation. Linear regression analysis of a cross-disorder PRS at p-value threshold <0.20 (Cross-PRS_0.20_) and DNA methylation of variable CpGs revealed a significantly skewed p-value distribution. Models was adjusted for population stratification, cell counts, gender, age at time of biosampling, maternal education and smoking. P-value from a pkstest.

**Figure S5:** Variable CpGs (vCpGs) that associate with the Cross-Disorder PRS (black circle) do not significantly overlap with vCpGs that associate with child abuse/neglect (grey circle). P-value is derived from a one-tailed Fisher exact test, numbers represent vCpGs associated with Cross-Disorder PRS child abuse/neglect.

**Table S1**:

| **PC10 and NFP treatment-associated vCpGs** | **p-value** | **Adj p-value** |
| --- | --- | --- |
| synapse assembly | 4.70E-17 | 2.21E-13 |
| neurogenesis | 4.49E-16 | 5.49E-13 |
| peripheral nervous system development | 4.02E-16 | 5.49E-13 |
| determination of left/right asymmetry in nervous system | 2.31E-15 | 1.36E-12 |
| autonomic nervous system development | 8.16E-16 | 5.49E-13 |
| ganglion development | 7.21E-16 | 5.49E-13 |
| stomatogastric nervous system development | 7.21E-16 | 5.49E-13 |
| nervous system development | 7.21E-16 | 5.49E-13 |
| neural tube development | 4.26E-15 | 2.23E-12 |
| central nervous system development | 2.16E-13 | 1.01E-10 |

| **PC1 and CAN-associated vCpGs** | **p-value** | **Adj p-value** |
| --- | --- | --- |
| protein phosphorylation | 7.06E-05 | 0.10 |
| positive regulation of T cell differentiation | 0.000155847 | 0.10 |
| Hemopoiesis | 0.000184287 | 0.10 |
| T cell differentiation | 0.000242553 | 0.10 |
| inflammatory response | 0.00096767 | 0.19 |
| liver development | 0.0006642 | 0.19 |
| positive regulation of protein ubiquitination | 0.000833824 | 0.19 |
| neutrophil degranulation | 0.002479537 | 0.28 |
| nuclear-transcribed mRNA catabolism | 0.002271739 | 0.28 |
| regulation of transcription from RNA polymerase II promoter | 0.013689801 | 0.49 |
| **PC2 and CAN-associated vCpGs** | **p-value** | **Adj p-value** |
| regulation of transcription by transcription factor localization | 9.96E-05 | 0.044 |
| regulation of transcription involved in exit from mitosis | 1.21E-04 | 0.044 |
| regulation of antisense RNA transcription | 1.27E-04 | 0.044 |
| regulation of DNA-templated transcription, termination | 1.47E-04 | 0.044 |
| regulation of transcription from RNA polymerase III promoter | 1.48E-04 | 0.044 |
| regulation of transcription involved in G1/S transition of mitotic cell cycle | 1.63E-04 | 0.044 |

**Table S2:**

**Table S3:**

| **Rank** | **CpG Identifier** | **Unadjusted p-value** |
| --- | --- | --- |
| 1 | cg03440944 | 1.47776E-06 |
| 2 | cg00115288 | 3.82537E-06 |
| 3 | cg16204559 | 8.35849E-06 |
| 4 | cg04702509 | 1.38023E-05 |
| 5 | cg21161138 | 1.60793E-05 |
| 6 | cg01498249 | 1.72501E-05 |
| 7 | cg04194840 | 2.74992E-05 |
| 8 | cg07784872 | 3.18013E-05 |
| 9 | cg11497458 | 3.30141E-05 |
| 10 | cg04718853 | 3.35668E-05 |
| 11 | cg09501047 | 3.55598E-05 |
| 12 | cg08889930 | 3.82525E-05 |
| 13 | cg12218389 | 4.55083E-05 |
| 14 | cg26364091 | 4.88963E-05 |
| 15 | cg18246298 | 5.04457E-05 |
| 16 | cg04453471 | 5.22257E-05 |
| 17 | cg27057329 | 5.50795E-05 |
| 18 | cg17166262 | 6.14106E-05 |
| 19 | cg16310491 | 6.24185E-05 |
| 20 | cg16911214 | 6.48386E-05 |
| 21 | cg07696699 | 6.60701E-05 |
| 22 | cg11796827 | 6.62743E-05 |
| 23 | cg25350097 | 6.76811E-05 |
| 24 | cg14072027 | 6.8371E-05 |
| 25 | cg05158854 | 6.8955E-05 |
| 26 | cg00806319 | 7.08339E-05 |
| 27 | cg24090911 | 7.18812E-05 |
| 28 | cg18984103 | 7.32025E-05 |
| 29 | cg03445800 | 7.43317E-05 |
| 30 | cg02556649 | 7.57178E-05 |
| 31 | cg04739149 | 7.61563E-05 |
| 32 | cg08677488 | 8.10427E-05 |
| 33 | cg13276570 | 9.20088E-05 |
| 34 | cg07423149 | 9.41225E-05 |
| 35 | cg00035316 | 9.42495E-05 |
| 36 | cg18669186 | 9.51754E-05 |
| 37 | cg06178299 | 9.98219E-05 |
| 38 | cg05713486 | 0.000103396 |
| 39 | cg03003394 | 0.000107179 |
| 40 | cg06096677 | 0.000112694 |
| 41 | cg07576363 | 0.000117276 |
| 42 | cg13291296 | 0.000126653 |
| 43 | cg09859659 | 0.000130248 |
| 44 | cg07519229 | 0.000131523 |
| 45 | cg14580211 | 0.000136013 |
| 46 | cg26099045 | 0.00014577 |
| 47 | cg24124079 | 0.000147495 |
| 48 | cg02577773 | 0.000147599 |
| 49 | cg15484808 | 0.000152556 |
| 50 | cg26946769 | 0.000153118 |
| 51 | cg10458392 | 0.000153197 |
| 52 | cg00155846 | 0.000155878 |
| 53 | cg17115737 | 0.000159982 |
| 54 | cg13491139 | 0.000170223 |
| 55 | cg22737154 | 0.000171657 |
| 56 | cg24251035 | 0.000172405 |
| 57 | cg08396193 | 0.000175419 |
| 58 | cg00164678 | 0.000176437 |
| 59 | cg13268603 | 0.000178265 |
| 60 | cg21451906 | 0.00018763 |
| 61 | cg19399532 | 0.00018979 |
| 62 | cg22228988 | 0.000206791 |
| 63 | cg13519452 | 0.000207447 |
| 64 | cg06938101 | 0.000211511 |
| 65 | cg20600845 | 0.000213477 |
| 66 | cg02229607 | 0.000214598 |
| 67 | cg15500259 | 0.000218136 |
| 68 | cg06123807 | 0.000225231 |
| 69 | cg27451610 | 0.000227605 |
| 70 | cg26881591 | 0.000238522 |
| 71 | cg18190861 | 0.000240368 |
| 72 | cg01062116 | 0.000245058 |
| 73 | cg23907053 | 0.000252962 |
| 74 | cg15252429 | 0.000260863 |
| 75 | cg16467775 | 0.000270673 |
| 76 | cg02308712 | 0.000283958 |
| 77 | cg24046474 | 0.000298069 |
| 78 | cg26153954 | 0.000304944 |
| 79 | cg11073381 | 0.000311493 |
| 80 | cg12119029 | 0.000321482 |
| 81 | cg12745160 | 0.000321746 |
| 82 | cg05650559 | 0.000329552 |
| 83 | cg23912266 | 0.000340073 |
| 84 | cg06369785 | 0.000340683 |
| 85 | cg01934638 | 0.000342554 |
| 86 | cg18099070 | 0.000347044 |
| 87 | cg02800810 | 0.000357537 |
| 88 | cg01384290 | 0.000358305 |
| 89 | cg13844248 | 0.000363727 |
| 90 | cg01069451 | 0.000363922 |
| 91 | cg12572827 | 0.00037276 |
| 92 | cg13008492 | 0.000374201 |
| 93 | cg24727216 | 0.000381804 |
| 94 | cg03625911 | 0.000394407 |
| 95 | cg01058070 | 0.000397203 |
| 96 | cg06615840 | 0.000397849 |
| 97 | cg10868715 | 0.000401632 |
| 98 | cg15065877 | 0.000406072 |
| 99 | cg26518580 | 0.000413804 |
| 100 | cg08129331 | 0.000416546 |

**Table S4:**

| **Transcription Factor** | **Cell type/Cell Line** | **P-value for enrichment** | **Adjusted P-value** |
| --- | --- | --- | --- |
| MITF | MELANOMA | 7.53634E-33 | 4.86094E-30 |
| ZNF217 | MCF-7 | 6.2387E-30 | 2.01198E-27 |
| FOXA2 | HepG2 | 6.2357E-25 | 1.00551E-22 |
| TFAP2C | MCF-7 | 8.61187E-25 | 1.11093E-22 |
| EGR1 | ERYTHROLEUKEMIA | 5.30676E-23 | 5.70477E-21 |
| TCF4 | U87 | 3.68498E-21 | 2.6409E-19 |
| HNF4A | HepG2 | 1.14496E-19 | 6.15418E-18 |
| PAX3-FKHR | RHABDOMYOSARCOMA | 2.97527E-18 | 1.27936E-16 |
| STAT3 | U87 | 6.01195E-18 | 2.42357E-16 |
| DROSHA | HELA | 1.03076E-17 | 3.91083E-16 |
| SCL | MEGAKARYOCYTES | 1.28665E-17 | 4.6105E-16 |
| KDM2B | K562 | 3.17287E-16 | 9.74524E-15 |
| **AR** | PC3 | 2.10956E-15 | 6.18483E-14 |
| KDM2B | SUP-B15 | 2.63747E-15 | 7.0882E-14 |
| CTNNB1 | HCT116 | 3.67022E-15 | 9.46916E-14 |
| AHR | MCF-7 | 5.41114E-15 | 1.34238E-13 |
| ARNT | MCF-7 | 2.8734E-14 | 6.46781E-13 |
| LXR | THP-1 | 4.58589E-14 | 9.85966E-13 |
| GATA1 | MEGAKARYOCYTES | 1.95371E-13 | 4.06497E-12 |
| SMAD4 | A2780 | 5.38882E-13 | 1.05327E-11 |
| TP63 | HFKS | 2.84547E-11 | 5.2438E-10 |
| KDM2B | JURKAT | 3.93844E-11 | 6.86565E-10 |
| CTCF | Bcells | 4.32963E-10 | 7.34897E-09 |
| UBF1/2 | HMEC-DERIVED | 8.38687E-10 | 1.38706E-08 |
| RACK7 | MCF-7 | 1.60752E-09 | 2.52891E-08 |
| RUNX2 | PCA | 2.02885E-09 | 3.11573E-08 |
| GATA2 | MEGAKARYOCYTES | 2.3131E-09 | 3.46966E-08 |
| SPI1 | K562 | 2.72997E-09 | 4.00189E-08 |
| CREB1 | LNCaP-abl | 3.04872E-09 | 4.36984E-08 |
| KDM2B | DND41 | 5.72108E-09 | 8.02195E-08 |
| **AR** | PROSTATE CANCER | 6.52203E-09 | 8.95045E-08 |
| SMAD3 | EPCs | 1.06559E-08 | 1.43189E-07 |
| KLF4 | ESCs | 1.44173E-08 | 1.85983E-07 |
| TP63 | EP156T | 3.94787E-08 | 4.71551E-07 |
| PPAR | NCI-H1993 | 5.38568E-08 | 6.31594E-07 |
| SMAD2/3 | EPCs | 1.13536E-07 | 1.30769E-06 |
| WT1 | PODOCYTE | 1.39651E-07 | 1.58026E-06 |
| TCF21 | SMOOTH MUSCLE | 1.50852E-07 | 1.64914E-06 |
| ELK3 | HUVEC | 1.50852E-07 | 1.64914E-06 |
| PIAS1 | VCAP | 2.83214E-07 | 3.04455E-06 |
| VDR | THP-1 | 5.21149E-07 | 5.33557E-06 |
| VDR | THP-1 | 7.59108E-07 | 7.65039E-06 |
| SA1 | ERYTHROID | 7.84814E-07 | 7.78777E-06 |
| SMAD3 | ESCs | 1.72682E-06 | 1.68758E-05 |
| PRDM14 | ESCs | 1.84822E-06 | 1.77926E-05 |
| KDM2B | SIL-ALL | 2.23404E-06 | 2.11905E-05 |
| KLF6 | PDAC-Cell Line | 4.76066E-06 | 4.38661E-05 |
| VDR | LCL-AND-THP1 | 5.2344E-06 | 4.7552E-05 |
| EZH2 | EOC | 5.60433E-06 | 5.02055E-05 |
| P300 | ESCs | 6.0936E-06 | 5.2405E-05 |
| KLF5 | YYC3 | 6.0936E-06 | 5.2405E-05 |
| RUNX | JUKART | 9.28448E-06 | 7.87959E-05 |
| CTCF | ERYTHROID | 9.90397E-06 | 8.29618E-05 |
| TCF3/E2A | JUKARTE6-1 | 1.02346E-05 | 8.46326E-05 |
| PPARD | MYOFIBROBLAST | 1.30486E-05 | 0.000106536 |
| FOXM1 | Hek293 flp-in | 1.62329E-05 | 0.000130878 |
| **AR** | VCAP | 1.97108E-05 | 0.000154453 |
| OCT4 | ESCs | 2.01147E-05 | 0.000154453 |
| KLF4 | PDAC-Cell Line | 2.01147E-05 | 0.000154453 |
| P300 | ZR-75-30cells | 2.01147E-05 | 0.000154453 |
| SA1 | Bcells | 3.18312E-05 | 0.000238734 |
| OCT4 | PROSTATE | 7.71993E-05 | 0.000559478 |
| E2A | RAMOS-Cell line | 9.56986E-05 | 0.00068584 |
| **NR3C1** | MCF10A | 0.000113733 | 0.000806126 |
| TBL1 | 293T | 0.000118315 | 0.000829489 |
| VDR | THP-1 | 0.000160207 | 0.001099295 |
| ESR2 | MCF-7 | 0.000178417 | 0.001192936 |
| FOXM1 | MCF-7 | 0.000179403 | 0.001192936 |
| TP53 | IMR90 | 0.000195835 | 0.001288912 |
| TP53 | U2OS | 0.000270621 | 0.001745507 |
| CREB1 | LNCaP | 0.000269146 | 0.001745507 |
| ERG | JURKAT | 0.000334831 | 0.002138274 |
| SMAD4 | EPCs | 0.000399502 | 0.002526261 |
| TRIM28 | HEK293 | 0.00052148 | 0.003265579 |
| TOP2B | MCF-7 | 0.000586712 | 0.003604088 |
| TCF12/HEB | JUKARTE6-1 | 0.000708183 | 0.004309227 |
| CSB | FIBROBLAST | 0.001023588 | 0.006170226 |
| RELA | FIBROSARCOMA | 0.001060229 | 0.006331922 |
| MYCN | NEUROBLASTOMA | 0.001225704 | 0.007253021 |
| **ESR1** | MCF-7 | 0.002110707 | 0.012376416 |
| KDM2B | HPB-ALL | 0.002454266 | 0.014133944 |
| CJUN | BT549 | 0.002900243 | 0.016409271 |
| NCOR1 | K562 | 0.002900243 | 0.016409271 |
| GBX2 | PC3 | 0.003259678 | 0.018282541 |
| NFYA | K562 | 0.003418228 | 0.019006528 |
| **AR** | LNCaP-1F5 | 0.004018121 | 0.022058609 |
| EWS-FLI1 | SK-N-MC | 0.004223164 | 0.022890261 |
| FOXA1 | LNCaP-1F5 | 0.004710875 | 0.025111689 |
| WDR5 | LNCAP | 0.004925376 | 0.026039898 |
| SMRT | Bcells | 0.005508564 | 0.028886372 |
| SCL | HPC-7 | 0.006471646 | 0.033662995 |
| RXR | LS180 | 0.007391659 | 0.037381034 |
| ERA | ENDOMETRIOID-ADENOCARCINOMA | 0.007476207 | 0.037381034 |
| UBF1/2 | HMECs | 0.00747297 | 0.037381034 |
| SMC4 | HELA | 0.008669916 | 0.042045832 |
| NFYB | K562 | 0.008669916 | 0.042045832 |
| ELF3 | PDAC-Cell Line | 0.008669916 | 0.042045832 |

**Table S5:**

| **Gene ID** | **Transcription factor** | **Z score** | **p-value** |
| --- | --- | --- | --- |
| ***NR3C1*** | **GCR-alpha** | **7.961** | **1.071E-14** |
| *SP1* | SP1 | 7.606 | 9.728E-14 |
| ***ESR1*** | **ESR1 (nuclear)** | **6.449** | **2.383E-10** |
| *POU5F1* | Oct-3/4 | 6.323 | 5.208E-10 |
| *STAT3* | STAT3 | 5.868 | 1.073E-08 |
| *GLI1* | GLI-1 | 4.53 | 0.00002036 |
| *RUNX2* | RUNX2 | 4.392 | 0.00002171 |
| *GLI3* | GLI-3 | 4.662 | 0.00002414 |
| *GATA4* | GATA-4 | 4.202 | 0.00004515 |
| *TCF7L2* | TCF7L2 (TCF4) | 4.003 | 0.00006994 |
| *DLX5* | DLX5 | 4.342 | 0.00008337 |
| ***PGR*** | **PR (nuclear)** | **3.984** | **0.00008371** |
| *ETS1* | ETS1 | 3.665 | 0.0002374 |
| *RARA* | RARalpha | 3.722 | 0.0002724 |
| *TFAP2A* | AP-2A | 3.583 | 0.0003523 |
| *LHX2* | LHX2 | 3.538 | 0.0003667 |
| *FOXA2* | HNF3-beta | 3.471 | 0.0004949 |
| *ZBTB20* | ZNF288 | 3.865 | 0.000503 |
| *NKX3-2* | BAPX1 | 3.609 | 0.0005444 |
| *NANOG* | NANOG | 3.303 | 0.0007122 |
| *FOXP2* | FOXP2 | 3.371 | 0.0007551 |
| *SMAD1* | SMAD1 | 3.338 | 0.001019 |
| *TAL1* | TAL1 | 3.221 | 0.001205 |
| *HNF4A* | HNF4-alpha | 3.082 | 0.001526 |
| *HOXA2* | HOXA2 | 3.368 | 0.001535 |
| *MEIS2* | MEIS2 | 3.329 | 0.003724 |
| ***NR3C1*** | **GCR-beta** | **3.097** | **0.003779** |
| *ONECUT2* | OC-2 | 3.358 | 0.004158 |
| *RXRA* | RXRA | 2.795 | 0.004297 |

**Table S6:**

| **Rank** | **CpG Identifier** | **Unadjusted p-value** |
| --- | --- | --- |
| 1 | cg01201279 | 1.85177E-05 |
| 2 | cg13775306 | 7.49203E-05 |
| 3 | cg03938110 | 7.86255E-05 |
| 4 | cg00281725 | 7.94661E-05 |
| 5 | cg20107346 | 8.0591E-05 |
| 6 | cg06458106 | 9.94869E-05 |
| 7 | cg13333304 | 0.000106192 |
| 8 | cg09423126 | 0.000111367 |
| 9 | cg03088958 | 0.000116695 |
| 10 | cg20956390 | 0.000121238 |
| 11 | cg14338345 | 0.000131267 |
| 12 | cg00777271 | 0.000136609 |
| 13 | cg00667751 | 0.000148734 |
| 14 | cg09427644 | 0.000151769 |
| 15 | cg11083807 | 0.000180456 |
| 16 | cg13172549 | 0.000204556 |
| 17 | cg16652855 | 0.000211533 |
| 18 | cg01997606 | 0.000217833 |
| 19 | cg05543520 | 0.000237678 |
| 20 | cg03277049 | 0.000244661 |
| 21 | cg21077957 | 0.000248077 |
| 22 | cg00632007 | 0.000250607 |
| 23 | cg20562823 | 0.000252237 |
| 24 | cg14751481 | 0.000257852 |
| 25 | cg16471600 | 0.000258678 |
| 26 | cg21249371 | 0.000265321 |
| 27 | cg07168232 | 0.00027417 |
| 28 | cg23022785 | 0.000276121 |
| 29 | cg14291663 | 0.000280943 |
| 30 | cg23867562 | 0.000290301 |
| 31 | cg03660034 | 0.000317599 |
| 32 | cg11553116 | 0.000327577 |
| 33 | cg00011482 | 0.000329631 |
| 34 | cg08578216 | 0.000330032 |
| 35 | cg10131075 | 0.000342324 |
| 36 | cg09143195 | 0.000348391 |
| 37 | cg04714638 | 0.000348814 |
| 38 | cg09244436 | 0.000352208 |
| 39 | cg21004633 | 0.000363643 |
| 40 | cg01417849 | 0.000368046 |
| 41 | cg24057218 | 0.000375504 |
| 42 | cg03236328 | 0.000380163 |
| 43 | cg13571479 | 0.000384258 |
| 44 | cg10862354 | 0.00039589 |
| 45 | cg05485062 | 0.000411338 |
| 46 | cg26037602 | 0.000423361 |
| 47 | cg16858587 | 0.000457638 |
| 48 | cg01298758 | 0.000458552 |
| 49 | cg20769177 | 0.000462615 |
| 50 | cg26112999 | 0.00046384 |
| 51 | cg04699104 | 0.000477363 |
| 52 | cg05581802 | 0.000490227 |
| 53 | cg17204337 | 0.000494513 |
| 54 | cg27606002 | 0.000517964 |
| 55 | cg17298543 | 0.000550906 |
| 56 | cg04439374 | 0.000558338 |
| 57 | cg04605681 | 0.000564777 |
| 58 | cg05202300 | 0.000574847 |
| 59 | cg19868364 | 0.000575247 |
| 60 | cg06048750 | 0.000583634 |
| 61 | cg05246645 | 0.00060064 |
| 62 | cg00244776 | 0.00063378 |
| 63 | cg19815271 | 0.000657705 |
| 64 | cg15727708 | 0.000673784 |
| 65 | cg16240816 | 0.000706387 |
| 66 | cg08126542 | 0.000716952 |
| 67 | cg06962918 | 0.000722523 |
| 68 | cg06829788 | 0.000738653 |
| 69 | cg11300809 | 0.000755915 |
| 70 | cg00945507 | 0.000779178 |
| 71 | cg04945457 | 0.000802867 |
| 72 | cg00159439 | 0.000813909 |
| 73 | cg27541691 | 0.000852186 |
| 74 | cg00014754 | 0.000859371 |
| 75 | cg01591025 | 0.000882423 |
| 76 | cg19155735 | 0.000924979 |
| 77 | cg11019771 | 0.000927539 |
| 78 | cg27404351 | 0.000945513 |
| 79 | cg00950244 | 0.000965801 |
| 80 | cg02678305 | 0.00098052 |
| 81 | cg05775862 | 0.001011131 |
| 82 | cg14545253 | 0.001026339 |
| 83 | cg02821464 | 0.001029927 |
| 84 | cg17216737 | 0.001033354 |
| 85 | cg04774364 | 0.001042505 |
| 86 | cg16622920 | 0.001071586 |
| 87 | cg17545652 | 0.001074396 |
| 88 | cg26924247 | 0.00111758 |
| 89 | cg21020871 | 0.001122875 |
| 90 | cg20490088 | 0.001125583 |
| 91 | cg13178170 | 0.001151416 |
| 92 | cg21668832 | 0.001155587 |
| 93 | cg20026346 | 0.001158403 |
| 94 | cg05503062 | 0.001163619 |
| 95 | cg06521145 | 0.001165808 |
| 96 | cg11557932 | 0.001175565 |
| 97 | cg01317029 | 0.001181574 |
| 98 | cg24773532 | 0.001182406 |
| 99 | cg15890794 | 0.001184712 |
| 100 | cg06190807 | 0.00118725 |

**Table S7:**

|  | **Control (n=98)** | **Nurse-visited (N=88)** | |
| --- | --- | --- | --- |
| **CpG Identifier (gene name)** | **Beta (SD)** | **Beta (SD)** | |
| cg01201279 (*LEKR1*) | 0.73 (0.082) | 0.67 (0.09) | |
| **Cohen's D** | 0.71 | | |
|  | **Power calculation** | | |
| **Alpha (p)** | 10E-7 | | |
| **Beta** | 0.95 | | |
| **Sample allocation ratio** | 1.1 | | |
| **Tails** | 2 | | |
| **Estimated sample size** | N=168 | | N=184 |

Linear regression analyses identified cg01201279 as the top ranked variably methylated CpG associated with maternal participation in the nurse family partnership (p=3.47E-05), independent of cell-counts, population stratification, smoking, current mental health, age at time of biosampling and a polygenic risk indicator for psychiatric diagnoses. We estimated Cohen’s D (Table S5) and used G*Power^1^ to calculate the projected sample size required to detect a genome-wide significant group difference between controls and nurse-visited participants.

**Figure S1:**


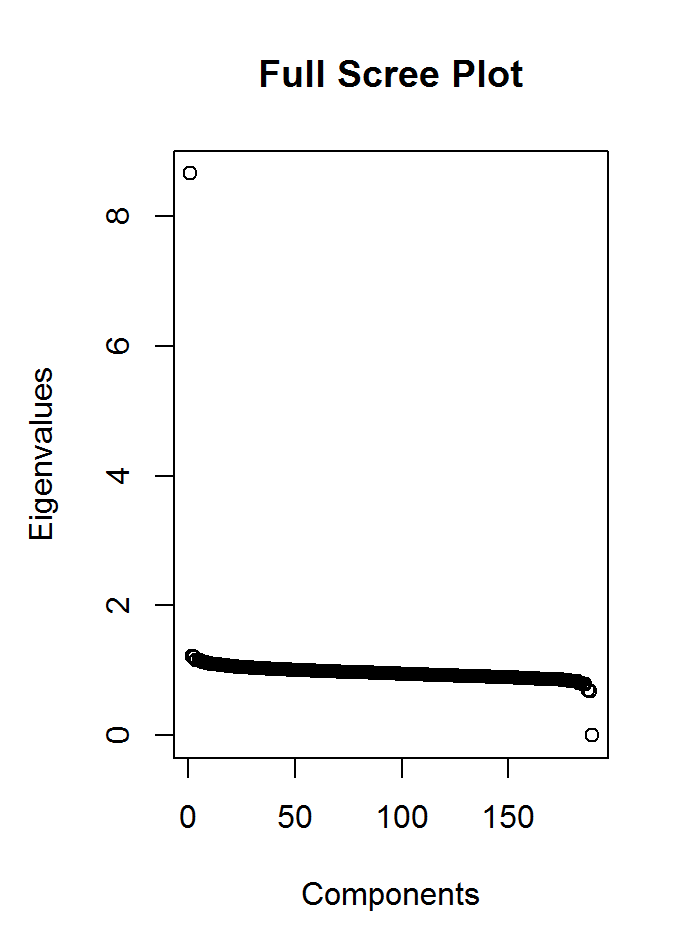

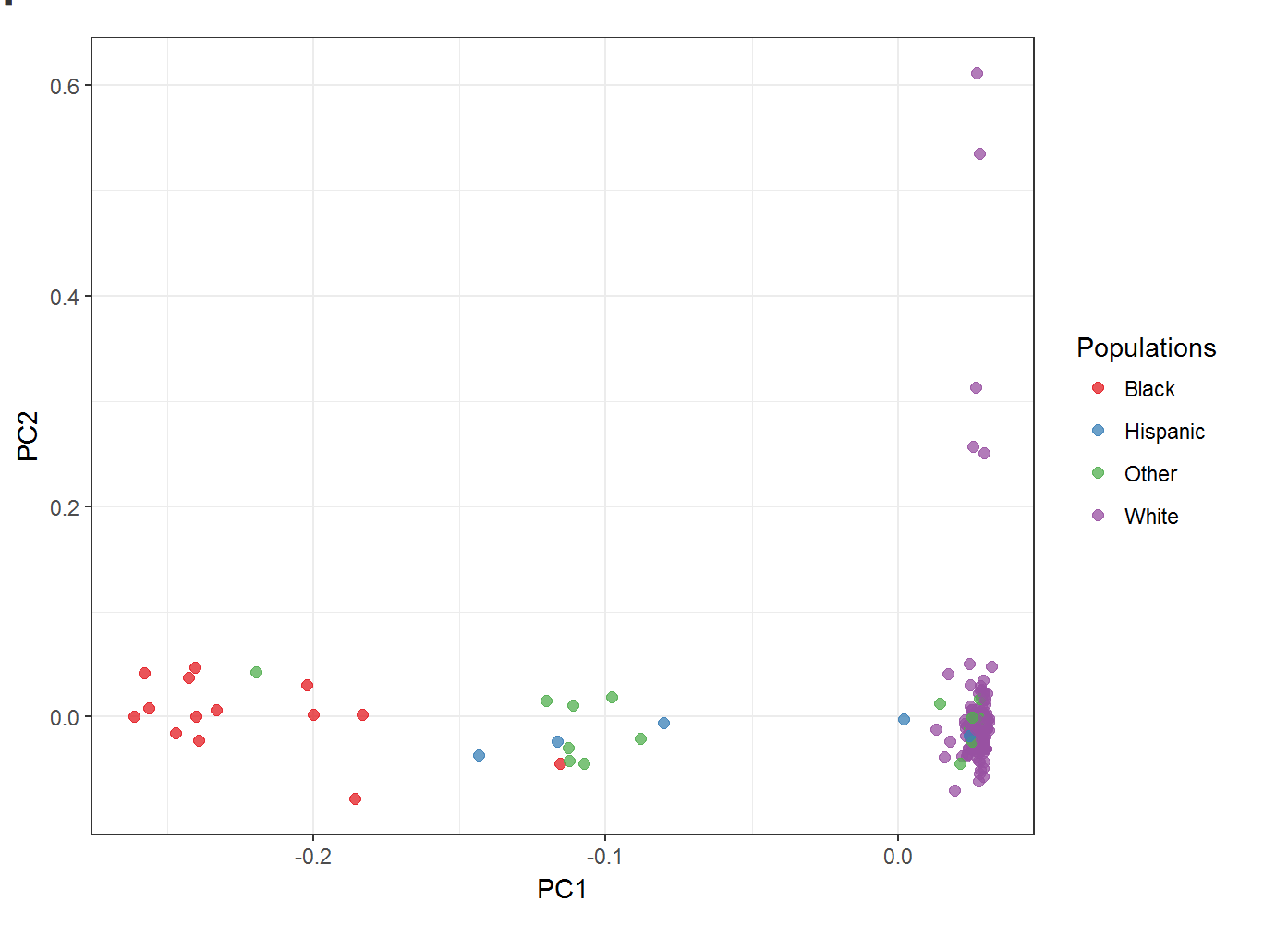


**Figure S2:**

**a.**


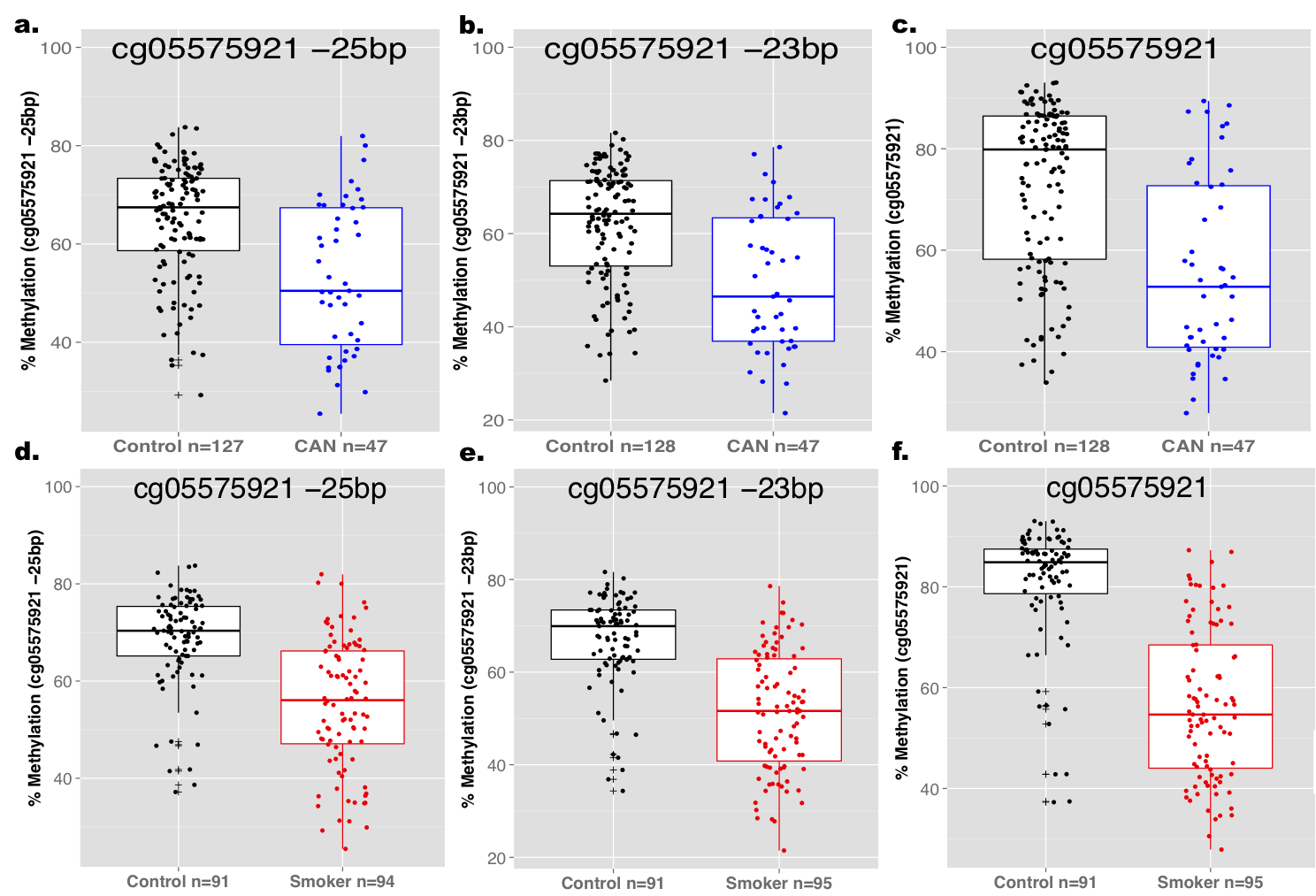


**Figure S3:**


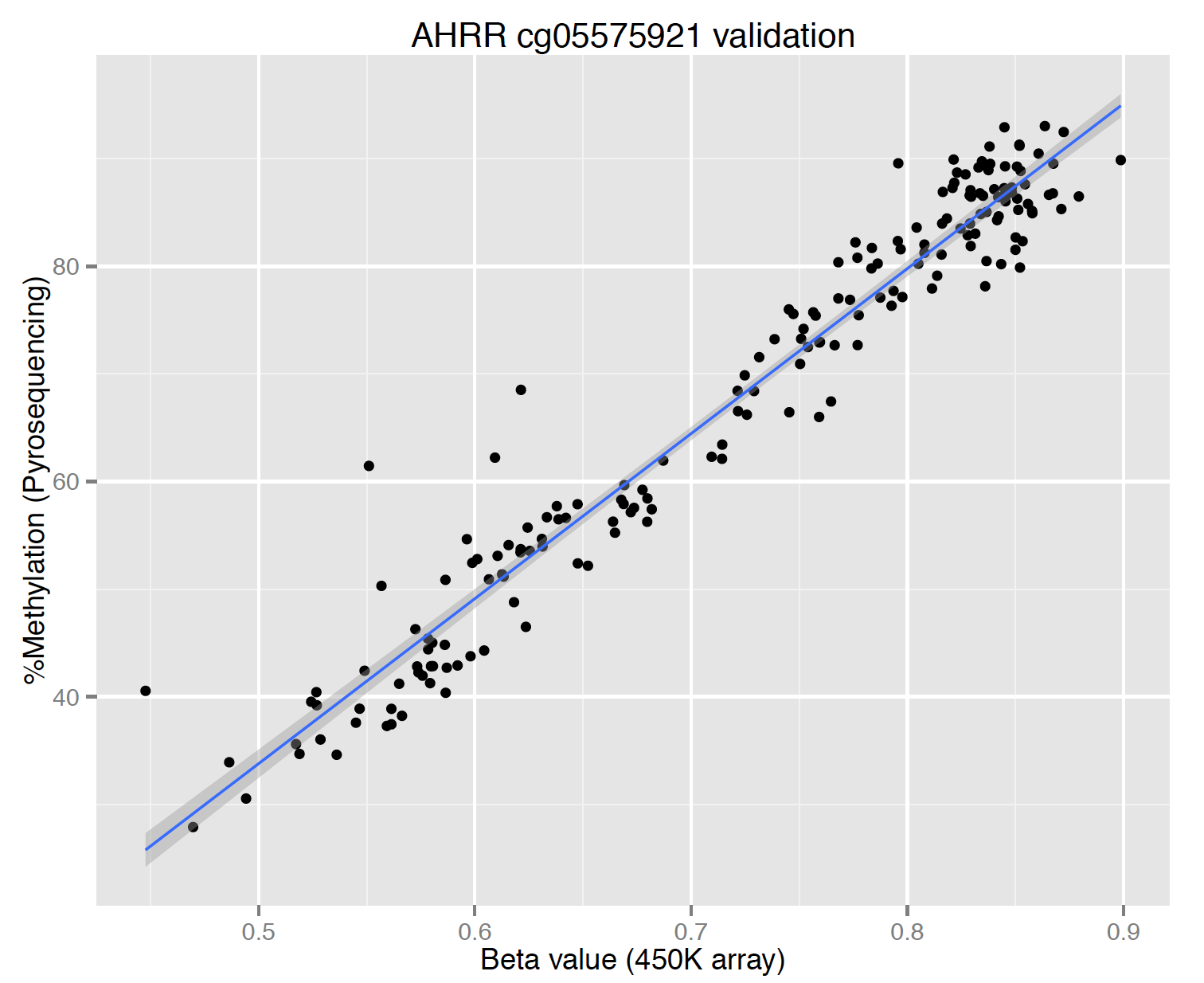

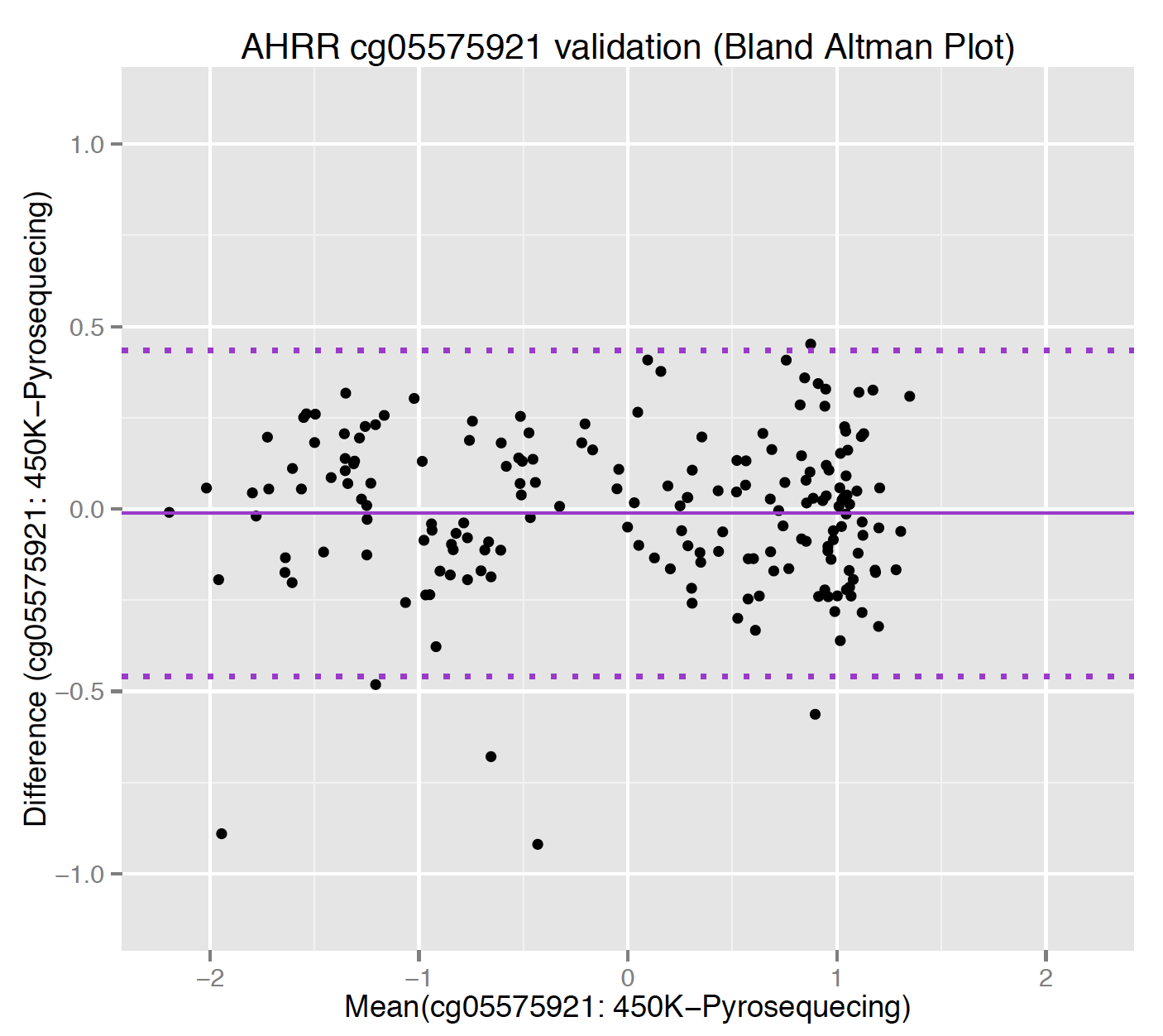


**r=0.974, p=3.87E-121**

**Figure S4:**


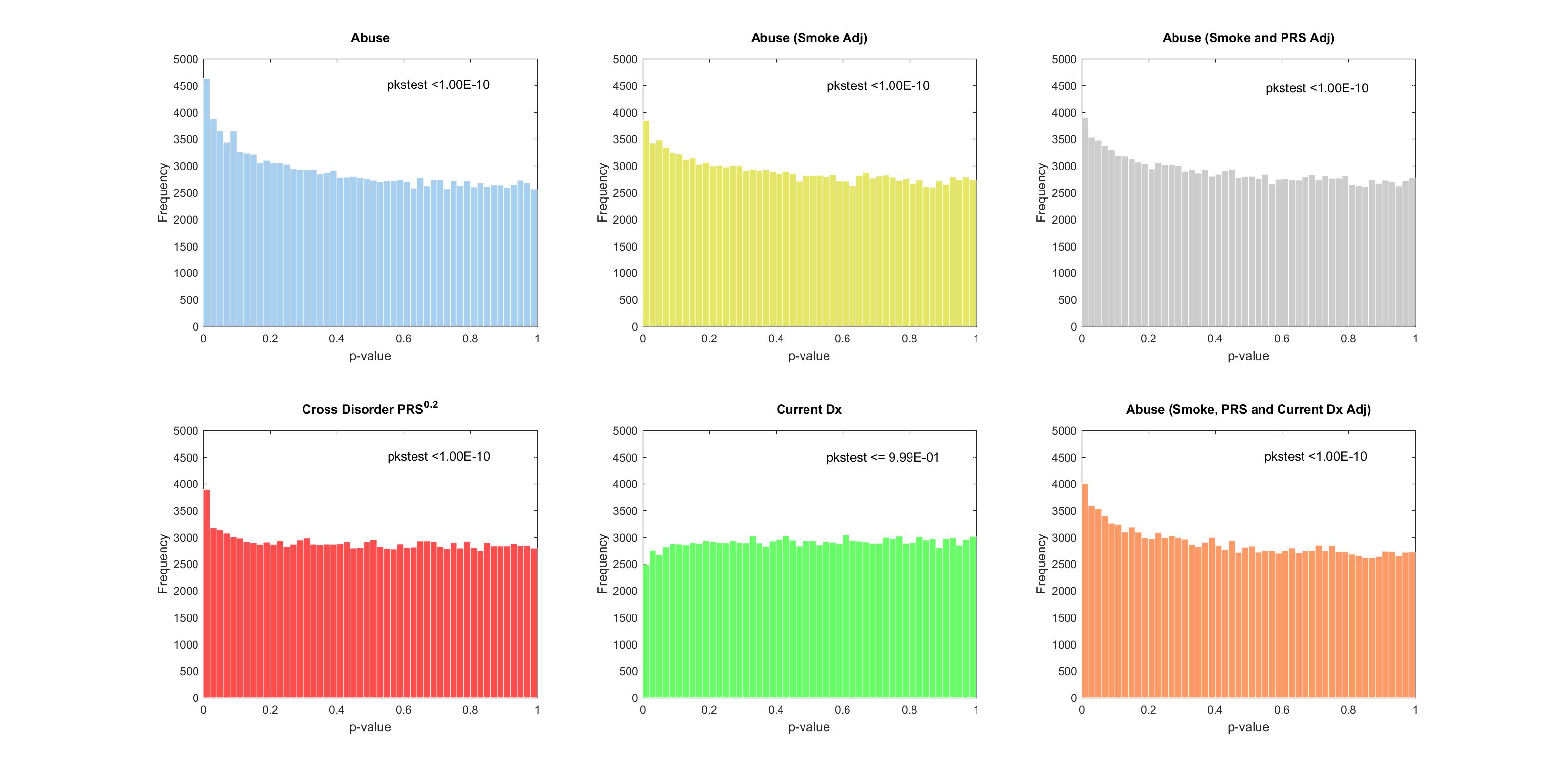


**Figure S5**


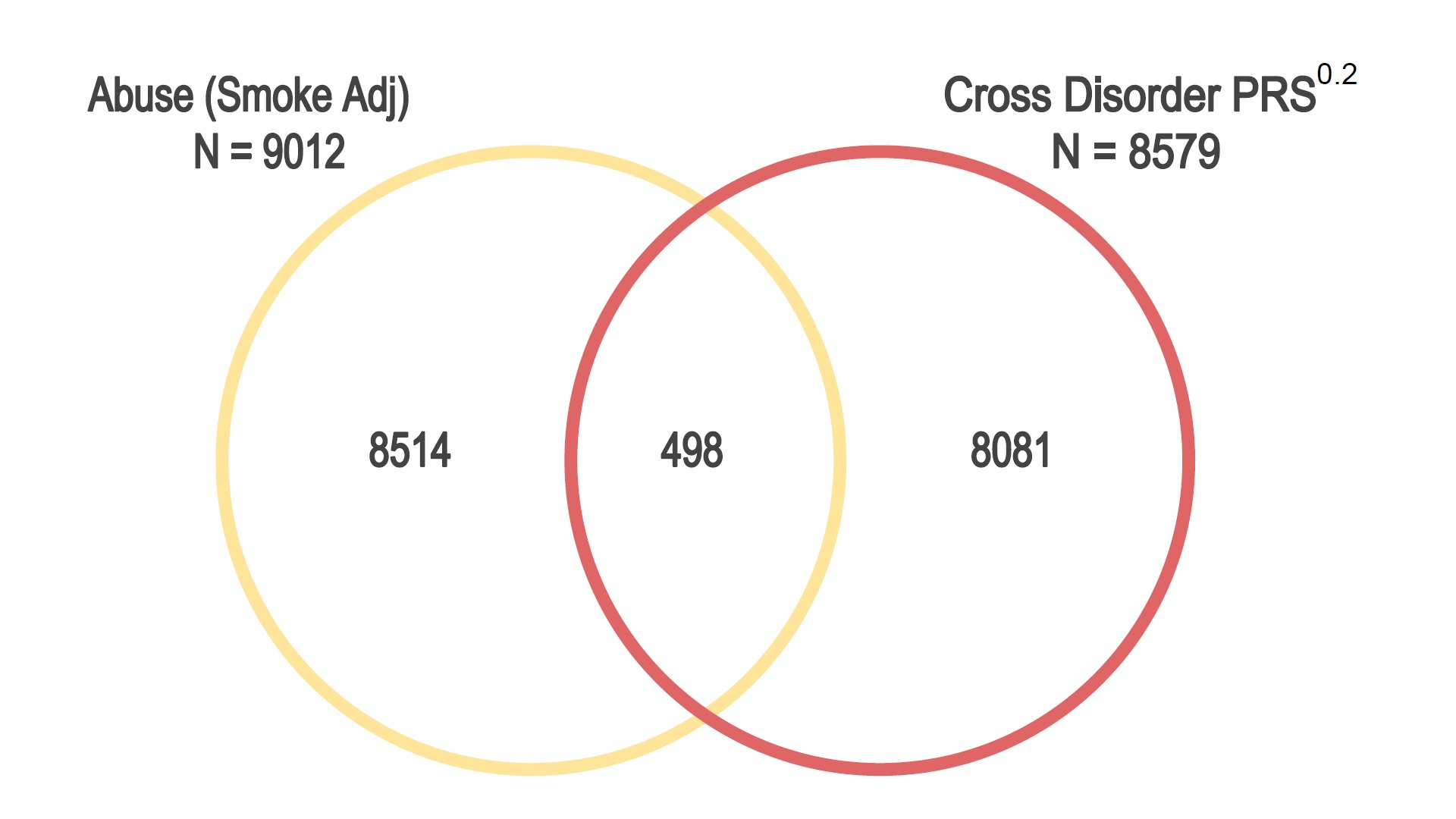


**P for overlap=0.117**

**Supplemental References:**

1. Faul F, Erdfelder E, Lang AG, Buchner A. G*Power 3: a flexible statistical power analysis program for the social, behavioral, and biomedical sciences. Behavior research methods. 2007;39(2):175-91.
